# Hyperspectral Classification of *Cyperus esculentus* Clones and Morphologically Similar Weeds

**DOI:** 10.3390/s20092504

**Published:** 2020-04-28

**Authors:** Marlies Lauwers, Benny De Cauwer, David Nuyttens, Simon R. Cool, Jan G. Pieters

**Affiliations:** 1 Department of Plants and Crops, Ghent University, 9000 Ghent, Belgium; marlies.lauwers@ugent.be (M.L.); Benny.decauwer@ugent.be (B.D.C.); 2 Technology and Food Science Unit, Flanders Research Institute for Agriculture, Fisheries and Food (ILVO), 9820 Merelbeke, Belgium; david.nuyttens@ilvo.vlaanderen.be (D.N.); simon.cool@ilvo.vlaanderen.be (S.R.C.)

**Keywords:** reflectance, logistic regression, partial least squares–discriminant analysis, random forest, yellow nutsedge, weed classification

## Abstract

*Cyperus esculentus* (yellow nutsedge) is one of the world’s worst weeds as it can cause great damage to crops and crop production. To eradicate *C. esculentus,* early detection is key—a challenging task as *it* is often confused with other *Cyperaceae* and displays wide genetic variability. In this study, the objective was to classify *C. esculentus* clones and morphologically similar weeds. Hyperspectral reflectance between 500 and 800 nm was tested as a measure to discriminate between (I) *C. esculentus* and morphologically similar *Cyperaceae* weeds, and between (II) different clonal populations of *C. esculentus* using three classification models: random forest (RF), regularized logistic regression (RLR) and partial least squares–discriminant analysis (PLS–DA). RLR performed better than RF and PLS–DA, and was able to adequately classify the samples. The possibility of creating an affordable multispectral sensing tool, for precise in-field recognition of *C. esculentus* plants based on fewer spectral bands, was tested. Results of this study were compared against simulated results from a commercially available multispectral camera with four spectral bands. The model created with customized bands performed almost equally well as the original PLS–DA or RLR model, and much better than the model describing multispectral image data from a commercially available camera. These results open up the opportunity to develop a dedicated robust tool for *C. esculentus* recognition based on four spectral bands and an appropriate classification model.

## 1. Introduction

*Cyperus esculentus* L. (yellow nutsedge) is a perennial C4 weed of the *Cyperaceae* family that originated from (sub) tropical areas and is listed as the sixteenth worst weed in the world [1]. In 1982, *C. esculentus* was detected for the first time in Limburg, the easternmost province of Flanders (northern part of Belgium) [2]. Since then, the species has moved in west through Flanders; it now covers an estimated agricultural area of 16,000 ha and is still spreading [3]. *Cyperus esculentus* is also spreading rapidly in Central Europe because of accidental introductions and subsequent expansion [4]. The species is hard to eradicate because of its enormous capacity for multiplying and spreading, and its low sensitivity to control measures [1]. *Cyperus esculentus* produces seeds and hard tubers at rhizome tips [5]. Tuber dispersal is generally regarded more important for the spread of this species than seed dispersal [6]; a single mother tuber is able to produce more than 1900 shoots and nearly 6900 tubers in an area of 3.2 m² in one year [7]. These tubers can stay dormant in the soil for several years; laboratory analysis showed a half-life of 5.7 months for tubers buried at 0.2 m [8], making eradication very hard [9]. Bohren and Wirth [10] summarized potential control methods for *C. esculentus* control, including cultural, mechanical and chemical methods; which is recommended depends on the infestation degree and spatial distribution. Initial small infestations can be controlled by removing all plant parts and infested soil, while heavy ones rely mostly on chemical weed control, or, in the worst case, require long fallows. Controlling *C. esculentus* is most effective when depleting existing tubers and preventing the formation of new ones [11] and relies on yearly repeated herbicide applications [12,13]. Pereira et al. [12] reviewed the suitability of different herbicides tested for combatting *C. esculentus* and designated the poor and temporary control, provided by most chemicals, as one of the reasons for failure. A combination of pre-emergence or preplant incorporated and postemergence herbicides have proven to be effective [14,15]. Another problem that farmers face when controlling *C. esculentus* is its genetic variability. There exist four wild varieties of *Cyperus esculentus*: var. *esculentus*, var. *heermannii*, var. *leptostachyus* Boeckeler and var. *macrostachyus* Boeckeler [16]. Mulligan and Junkins [17] stated that there exists evidence of significant genetic differences among *C. esculentus* populations and that these differences are relevant to the control of the species. De Cauwer et al. [3] observed large interclonal differences in herbicide sensitivity in Belgian *C. esculentus* clones. Additionally, although successful trials have been completed [13,18], eradication success greatly depends on early detection and treatment [19]. As young growth stages are more susceptible to chemical treatment [20,21,22], and misclassification can result in an enormous number of tubers, it is necessary to adequately and quickly determine this species. In addition, because of its risk to agriculture, farmers in Belgium are required by law to control *C. esculentus.* When *C. esculentus* is detected on a field, it is illegal to grow root, tuber or bulb crops and to remove infested soil; farmers are obliged to clean machinery when leaving an infested field and take appropriate control actions [23]. The European and Mediterranean Plant Protection Organization (EPPO) has included the weed on the list of invasive alien plants, against which action should be taken to prevent the spread within its member states [24]. 

However, *C. esculentus* is often confused with other *Cyperaceae*. Bearing in mind the species diversity of this family, and the implications this weed has for farmers, there is a strong need for a cheap and portable detection system. Reflectance spectroscopy is built on the idea that different plant species, or in extension, varieties, might induce distinct spectral features which can be used in species discrimination. It has proven to be able to distinguish between different weed-crop combinations [25,26,27,28,29,30,31,32], between different co-occurring species [33,34,35] and even between different clonal populations of one species [36]. Hyperspectral spectrometers measure reflectance with a very high spectral resolution and are able to detect small differences in reflectance. Hyperspectral sensors sensitive in the range of 400–900 nm have been used for classifying different varieties of maize [37]. A hand-held spectrometer with a leaf clip having a built-in integrating sphere is a good alternative to computer-based scatter corrections [38,39], limiting the time needed for preprocessing. As a result, the combination of a hand-held spectrometer with hyperspectral resolution and a leaf clip with an integrating sphere seems to be a suitable setup for recognizing *C. esculentus.* Although hyperspectral spectroradiometers are much cheaper than most imaging sensors, they are still expensive for farmers, especially when used for only one—very economically important—weed. Spectroradiometers with fewer wavelengths offer a more affordable solution.

In this study, the objective was to classify *C. esculentus* clones and morphologically similar weeds. To that end, hyperspectral reflectance was tested as a measure to discriminate between (I) *C. esculentus* and morphologically similar *Cyperacea* weeds, and between (II) different clonal populations of *C. esculentus*. The robustness of the models created for Experiment I was checked using two datasets (III): data of Experiment II and a dataset consisting of *C. esculentus* samples from an infested maize field in Lede, Belgium. To develop a low-cost tool for farmers in the future, a study was done to appoint particular wavelengths in Experiment I that are able to discriminate between *C. esculentus* and other weeds (IV). Classification results of this study were compared against simulated results from a commercially available multispectral camera with four spectral bands.

## 2. Materials and Methods

To determine the discriminatory ability of hyperspectral reflectance within the *Cyperaceae* family, two Experiments were performed. The first Experiment (I) was conducted in 2018 in Ghent, Belgium. Two morphologically similar species were selected that are often mistaken for *C. esculentus* and are frequently found in maize fields in Flanders: *Bolboschoenus maritimus* (L.) Palla (bayonet grass) and *Carex hirta* L. (hairy sedge). For *C. esculentus*, tubers of the botanical variety *Cyperus esculentus* var. *leptostachyus* were used; so far, the only *C. esculentus* variety present in Belgium [2,40]. Rhizomes of *B. maritimus* and *C. hirta* originated from locally collected species. Vegetative propagules were planted in 12 L pots. For each species, 24 individuals (one per pot) were planted in pot substrate provided with NPK (nitrogen, phosphorus and potassium) fertilizer (14:16:18). All pots were randomly placed in the greenhouse and periodically rearranged in a random fashion. Plants were watered once or twice a day depending on their needs. The summer of 2018 was abnormally sunny and warm with an average temperature of 19.8 °C and a total sunshine duration of 693.1 h [41], causing the temperature in the greenhouse to be above normal. Average normal temperature and total sunshine duration are 17.5 °C and 578.2 h, respectively.

**Figure 1:**
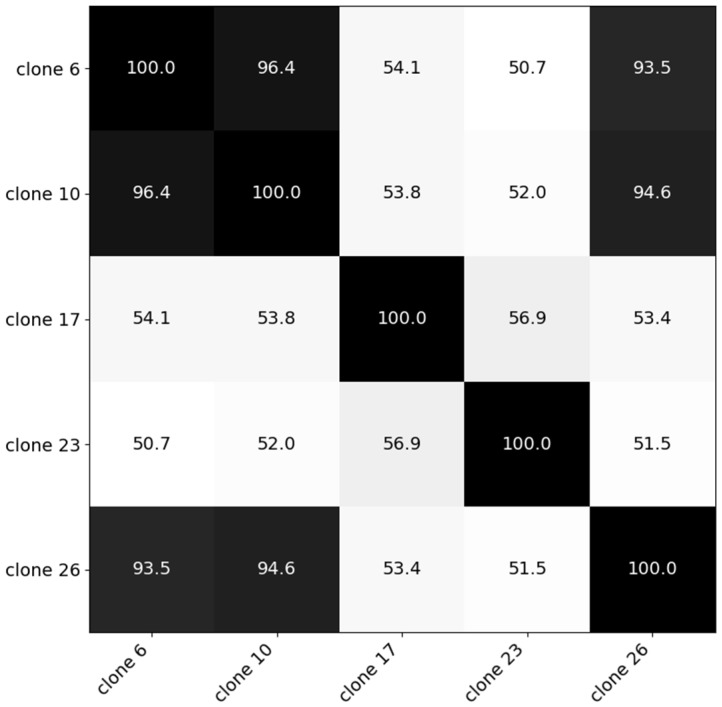
Similarity matrix of the five clones created using genetic data [42]. Clone 17; clone 23; and clones 6, 10 and 26 were selected from three distinct clusters.

The second Experiment was conducted in 2019 in Melle and investigated the hyperspectral distinctiveness of different *C. esculentus* clones and its impact on the model created in Experiment I. Clonal populations used in Experiment II, sampled by De Cauwer et al. [3], were found in Belgian maize fields and were morphologically and genetically distinct. In each 10 L pot, a tuber was planted. They were placed in open air and equally watered. For the clones in this trial a similarity matrix was created based on genetic distance [42] (Figure 1). Based on cluster analysis, five clones were selected from three distinct clusters. For each clone, four replications were available. Average temperature during the summer of 2019 was 19.1 °C and the total sunshine duration was 714.4 h [43]. 

For all experiments, hyperspectral reflectance measurements were executed using a spectrometer (FLAME-S-VIS-NIR-ES, Ocean Optics, Inc., Largo, FL, USA) with a range of 350–1000 nm and a spectral resolution of ±0.35 nm at 600 nm. An internal light source was used (HL-2000-HP-FHSA, Ocean Optics, Inc., Largo, FL, USA) together with a leaf clip (SPECTROCLIP-R, Ocean Optics, Inc., Largo, FL, USA). OceanView software (Ocean Optics, Inc., Largo, FL, USA) was transferred to a raspberry pi (Raspberry Pi 3 Model B Rev 1.2, 256 MB). All equipment was installed in a box (IP 55, Legrand Plastic Enclosures, Limoges, France) and powered by a 4500 mAh, 6S LiPo battery (Hacker Motor GmbH, Ergolding, Germany) and a 15,000 mAh Li-ion Voltcraft powerbank.

**Figure 2:**
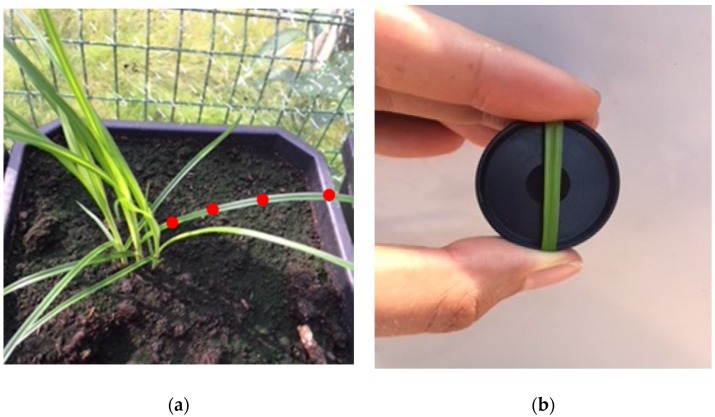
(**a**) Measurement spots for all *Cyperaceae* species; (**b**) positioning of a leaf blade on the reference.

The plants in Experiment I were measured once every one or two weeks. All twenty clones in Experiment II (5 clones × 4 replications) were measured six times over a period of two months. When possible, four measurements were taken on one leaf and averaged (Figure 2a). The species; date and time of measurement; pot number; amount of leaves; and for Experiment II, replication, were recorded. Twenty-four *C. esculentus* plants were sampled once in Lede in June of 2018 after thorough verification of species authenticity by a botanist. Leaves were measured destructively in order to position the sensor in such a way that it only captured leaf reflectance and not the reflectance of the black background reference. Figure 2b shows the black reference. The sensor measured only in the center of this reference. Measurements were executed on one leaf, so as not to hamper plant growth. Older (bigger) basal leaf blades were sampled because it was easier to position the sensor on them. Reflectance was calculated using Python (version 3.6.1) as

(1) $$R\left(\lambda \right)=\frac{X\left(\lambda \right)-DC\left(\lambda \right)}{WR\left(\lambda \right)-DC\left(\lambda \right)}$$

with X(λ) being the raw digital number (DN) values of a plant sample, DC(λ) the DN values of dark current and WR(λ) the DN values of the white reference, all measured with the leaf clip for a region around wavelength 𝜆. The white reference was measured using the spectralon provided by the manufacturer; the dark current was measured by switching off the shutter switch. Data gathered during measurements and reflectance data were stored together. In the next section the notation **X** will be used for the data matrix. **X** contains reflectance values with p columns for the spectral bands and n rows for the reflectance measurements.

Reflectance graphs were made for visual inspection of the measurements. There appeared to be a lot of noise near the edges. After visual inspection of the graphs, only reflectance values for wavelengths between 500 and 800 nm were used in the models described below. For most models, autoscaling was performed; i.e., the mean of each feature or column of **X** was subtracted from the original reflectance values and divided by the standard deviation. A Savitzky–Golay (SG) filter with varying window width, polynomial order and with or without a derivative, was tested together with autoscaling. 

To classify the different weed species or clone clusters, three models were selected that can handle high dimensional data combined with a low number of samples: random forest (RF), regularized logistic regression (RLR) and partial least squares–discriminant analysis (PLS–DA). RF was chosen for its ability to quantify feature importance [44,45], its resistance to overfitting [44] and its interpretability. RF has been successful in classifying vegetation with the use of hyperspectral data [46,47,48,49]. Regularized logistic regression has the advantage of producing class probabilities, while it is also resistant to overfitting and performs feature importance. RLR has been suggested to select hyperspectral bands [50] and build an inexpensive screening device [51]. PLS–DA finds its origin in chemometrics. It also produces class probabilities.

### 2.1. Random Forest (RF)

Random forest is an ensemble of decision tree classifiers [44]. For classification, a solution is found with a majority vote. Each tree is created using a bagged dataset consisting out of l predictors. Bagging, or bootstrap aggregation, creates new datasets by sampling features with replacement, and trains a classifier using each dataset. Splits in the tree are made using the Gini index:

(2) $$G=\overset{K}{\underset{k=1}{\sum }}p_{mk}*\left(1-p_{mk}\right)$$

with $p_{mk}$ being the fraction of class k at node m to the total samples and K the number of classes. A node with high purity, i.e., high or low values of $p_{mk}$, results in small values for G. With each split, a cut point s and a predictor j are obtained that minimize G. Random forests consider only l out of a total of p predictors when choosing this cutpoint. Often, $l=\sqrt{p}$ is chosen. This is done to reduce correlation among the trees; when all predictors are considered, all trees will look similar if there are a few very strong predictors, because all trees will select one of those as the first predictor. It is a nonparametric, nonlinear method.

An advantage of random forests is the ability to generate feature importance values. For each predictor, the drop in G is a measure of the importance; it is averaged over all trees and rescaled so the sum of all measures is one.

### 2.2. Regularized Logistic Regression (RLR)

Logistic regression fits a logistic function to estimate the probability of whether an event is happening or not; it has only two outcomes. The logistic function with only one variable is of the form:

(3) $$\varphi \left(z\right)=\frac{e^{z}}{1+e^{z}}$$

Multiple logistic regression uses not one, but multiple predictor variables and is of the form:

(4) $$\varphi \left(X\right)=\frac{e^{\beta_{0}+\beta_{1}X_{1}+\ldots +\beta_{p}X_{p}}}{1+e^{\beta_{0}+\beta_{1}X_{1}+\ldots +\beta_{p}X_{p}}}$$

with $\beta_{0},\ldots ,\beta_{p}$ being parameters of the model. The function has an outcome between zero or one; most of the time 0.5 is used as a threshold to make the classification. If more cost is involved with misclassification of one group, another threshold can be chosen. As these models can only distinguish between two groups, they cannot be used to classify multiple species. They can be used in a “one vs. one” (ovo) or “one vs. rest” (ovr) way. Using logistic regression with hyperspectral data means fitting many of parameters. A complex model can lead to overfitting. To avoid this, regularization can be applied. The method used here is called the lasso and combines regularization and feature selection. The cost function to be minimized with the lasso is

(5) $$-LL+C\overset{p}{\underset{j=1}{\sum }}\left|\beta_{j}\right|$$

with *C* a regularization parameter that needs to be optimized and LL the log likelihood. Lasso regularization adds a penalty term to force some of the parameters to zero. Large values of *C* create a large penalty term and will force estimates of $\beta_{j}$ to zero. When *C* is zero, no regularization is applied. With the optimal value for *C*, the $\beta_{j}$ parameters express feature importance. Regularization improves model test results and interpretation. RLR is a linear model.

### 2.3. Partial Least Squares–Discriminant Analysis (PLS–DA)

Partial least squares–discriminant analysis is the categorical variant of PLS-regression (PLS-R) [52]. In PLS-R a regression model is formed between **X** and **c**, in which **c** contains continuous numbers. In PLS–DA on the other hand, **c** contains numerical labels according to their group membership. The model correlates variation in the dataset with this group membership. Ruiz-Perez and Narasimhan [53] describe the method as a “supervised” version of principal component analysis as it achieves dimensionality reduction but with full awareness of the class labels. The number of latent variables is a hyperparameter of the model. 

For Experiment I, reflectance spectra of plants 1–17 were used to train all models, and those of plants 18–24 to test them. The total number of samples was different for each species; *B. maritimus*, *C. esculentus* and *C. hirta* had 35, 53 and 17 test samples, respectively. Sample size differed because vegetative material of *B. maritimus,* and especially *C. hirta*, had problems sprouting. Replications 1–3 and 4 were used to train and test models, respectively, for Experiment II, thereby obtaining a total of 30 test samples (1 replication × 5 clones × 6 times). A classification was made for both datasets using the three different models. 

For RLR the hyperparameter C was found using cross validation. One hundred $C^{-1}$ values in a logarithmic scale between $10^{-4}$ and $10^{4}$ were tested. The choice was made to perform ovr classification for interpretability. The β parameters were calculated using the “liblinear” solver [54] in Python, which is a coordinate descent algorithm and supports lasso regularization [55]. **X** was autoscaled. “f1_micro” was used in Python as the scoring function in RLR and RF, which is a global metric that includes precision and recall and is preferred with class imbalance. RF-results were also obtained using Python. The hyperparameters (Table 1) were again calculated using cross validation. The number of trees in the forest was set to 100. For Experiment II, the parameter class_weight was set to “balanced” in both RLR and RF. For other parameters, default settings were used. For the implementation of abovementioned models, the python package scikit-learn [56] was used. 

PLS–DA was performed using the PLS-toolbox (Eigenvector Research, Inc., Wenatchee, WA, USA) of Matlab (R2017b, The MathWorks Inc., Natick, MA, USA). Preprocessing was done using the model optimizer. Venetian blinds with 10 splits and blind thickness =1 was selected as the cross validation method. Q residuals reduced were plotted against Hotelling’s T² reduced to check for outliers. If no good reason was found (e.g., a remark saying equipment acted strange) to delete the measurement, they were kept in the model. Results of PLS–DA were obtained using the “Class Pred Most Probable” classification rule, meaning class predictions were those with the highest probability, even if the probability was low. Confusion matrices were created for all models.

In another test, data of Experiment II and data from sampled *C. esculentus* plants, present in an infested maize field, were used as inputs in the models that were created using the data from 2018 to test the robustness of the model (III). 

To test whether it would be possible to create a device sensitive to a small number of discriminatory wavelengths, one which would be able to make a good classification system, a new RLR model was created. First, reflectance spectra were resampled so the average wavelength interval was 10.2 nm. The wavelengths of the four β parameters with largest absolute value for *C. esculentus* were selected for a new logistic regression (IV). For this model, the solver was changed to “lbfgs,” as “liblinear” does not support logistic regression without regularization [55]. The same plants were used for training and testing the model as in Experiment I. Results were compared to a classification obtained from simulating a commercially available multispectral camera. The multispectral camera used was the Parrot Sequoia (Parrot SA, Paris, France), which has four spectral bands: green (550 nm), red (660 nm), red edge (735 nm) and near infrared (790 nm). All bands have a width of 40 nm except for the red edge filter, which has a width of 10 nm. This camera was used because it is not sensitive in blue wavelengths, which were discarded for high noise. The simulated reflectance was calculated by multiplying, for each band, the original measured spectra and the normalized sensitivity data, which were extracted from a graph provided by the manufacturer, and integrating the result, as was done by Gutiérrez et al. [57].

## 3. Results and Discussion

### 3.1. Classification Results (Experiments I and II)

**Figure 3:**
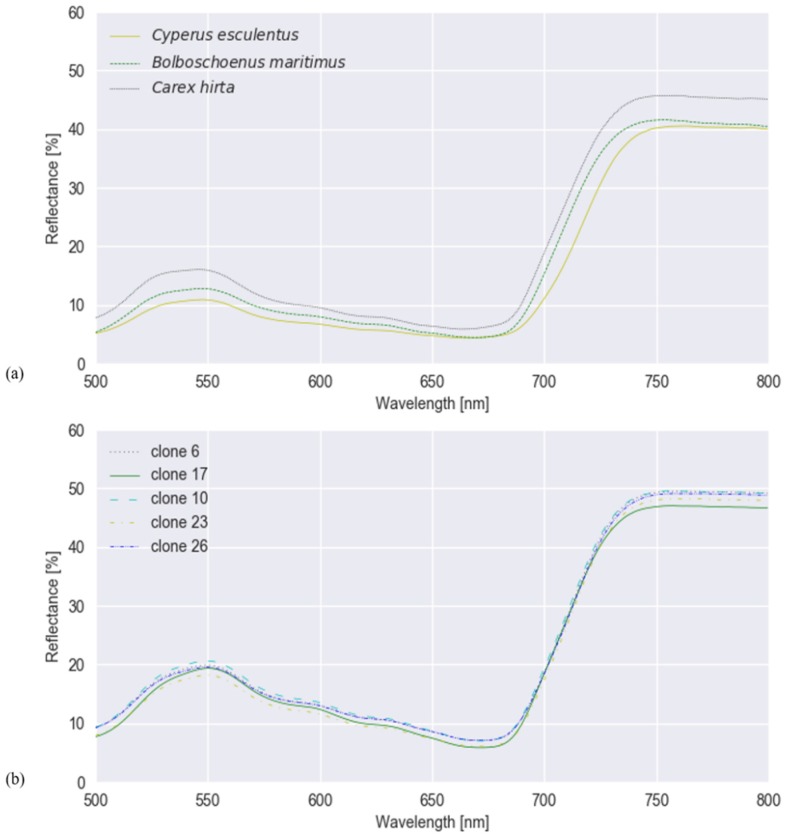
Mean spectral reflectance values of (**a**) *Cyperus esculentus*, *Bolboschoenus maritimus* and *Carex hirta* of experiment I, and (**b**) the five clones of *Cyperus esculentus* of experiment II, smoothed with Savitzky–Golay.

**Figure 4:**
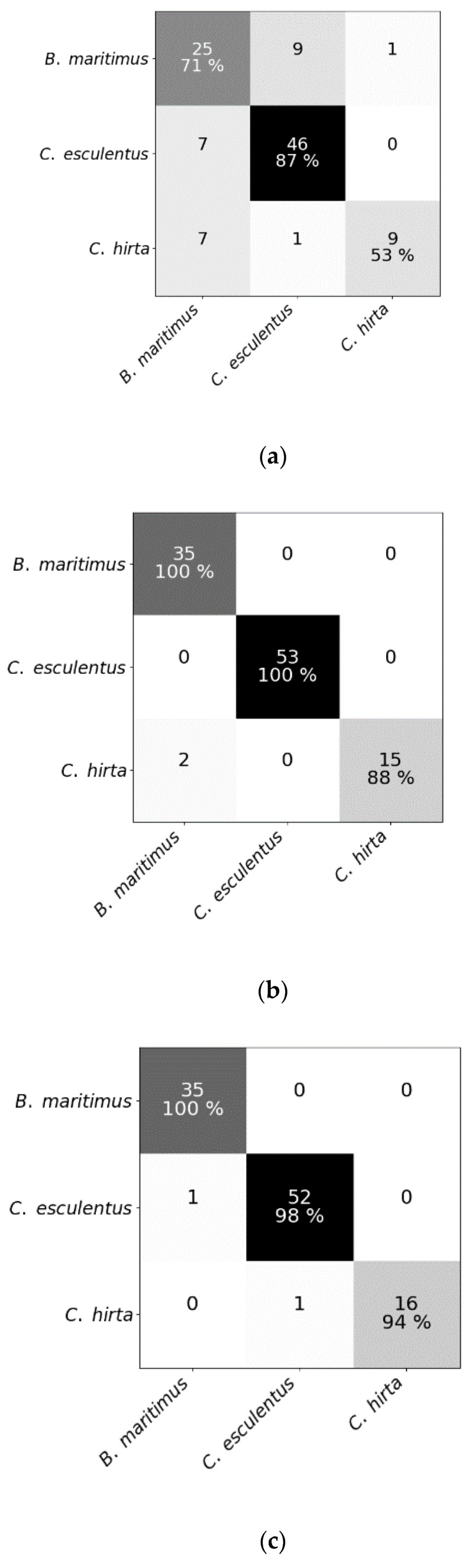
Confusion matrices of (**a**) the random forest classifier, (**b**) the regularized logistic regression and (**c**) partial least squares–discriminant analysis for the test dataset comprising reflectance data of *Cyperus esculentus, Bolboschoenus maritimus* and *Carex hirta* (Experiment I).

**Figure 5:**
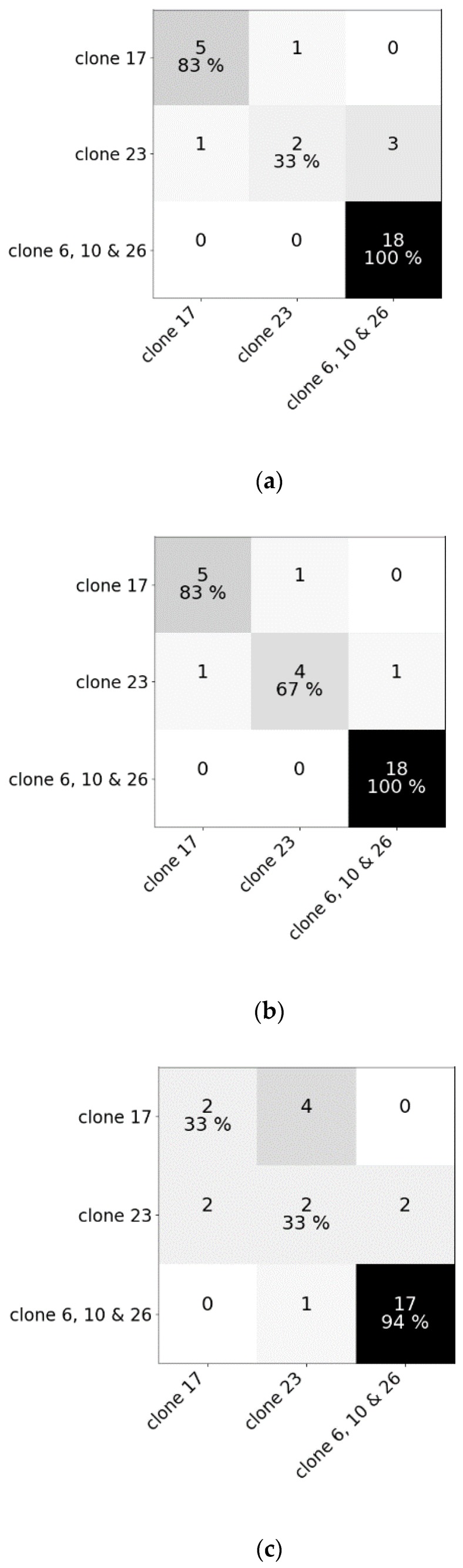
Confusion matrices of (**a**) the random forest classifier, (**b**) the regularized logistic regression and (**c**) partial least squares–discriminant analysis for the test dataset comprising reflectance data of five clones of *Cyperus esculentus* (Experiment II).

Mean spectral reflectances of all classes in both experiments are plotted in Figure 3. To test whether hyperspectral reflectance is a good measure to distinguish *C. esculentus* from morphologically similar weeds (I) and between different clonal populations (II), hyperspectral reflectance signatures were collected and a classification was done using three different models; RLR, RF and PLS–DA. Confusion matrices were created for each model type (Figure 4 and Figure 5).

Results of RF for this experiment were obtained using SG with window length 103 and polynomial order 1. Results of RLR were obtained using SG with window length 15, polynomial order 1 and a first order derivative together with autoscaling. Results of PLS–DA were obtained with SG with window length 41, polynomial order 3 and a second order derivative, and also autoscaling. Four latent variables were selected. RLR and PLS–DA classified most measurements of Experiment I correctly; only two measurements were misclassified. One of the samples that was misclassified in RLR, was also misclassified in RF but not in PLS–DA. The two misclassified samples in PLS–DA were also incorrectly classified in RF. Hyperparameter values of RF are given in Table 1. Based on the number of correctly classified samples, RLR and PLS–DA outperformed RF. The accuracy of training and test set deviated much more for RF than for the other models, indicating overfitting, although the previously mentioned parameters (Table 1) were tuned in order to decrease the risk of overfitting. The final goal was to distinguish between *C. esculentus* and other species, not to classify each individual into its corresponding species. Recall and precision [58] of *C. esculentus* in all matrices of Figure 4 should be close to 1. Misclassifying measurements of *B. maritimus* or *C. hirta* as *C. esculentus*, as is the case for RF and PLS–DA, can have unnecessary economic consequences for farmers and impact their choices of herbicide. The opposite also occurred in these two models, which might lead to farmers thinking they do not have a problem. Based on these figures, RLR is appointed the best model to handle *C. esculentus.*

For experiment II, the best results were acquired using RLR with 27 correctly classified samples out of 30. These results were obtained using SG with window length 55 and polynomial order 2. RF and PLS–DA classified 25 and 21 samples correctly, respectively. Hyperparameter values for RF of experiment II are also given in Table 1. Being able to classify these clones by genetic cluster shows that the morphological and genetic distinctions of the clones are translated into distinct spectral reflectances, creating the possibility to hyperspectrally detect intraspecific variability in genetic background and implement cluster-based chemical control measures. However, additional validation with a bigger dataset is recommended. For both experiments, RLR has proven to be most effective. 

**Figure 6:**
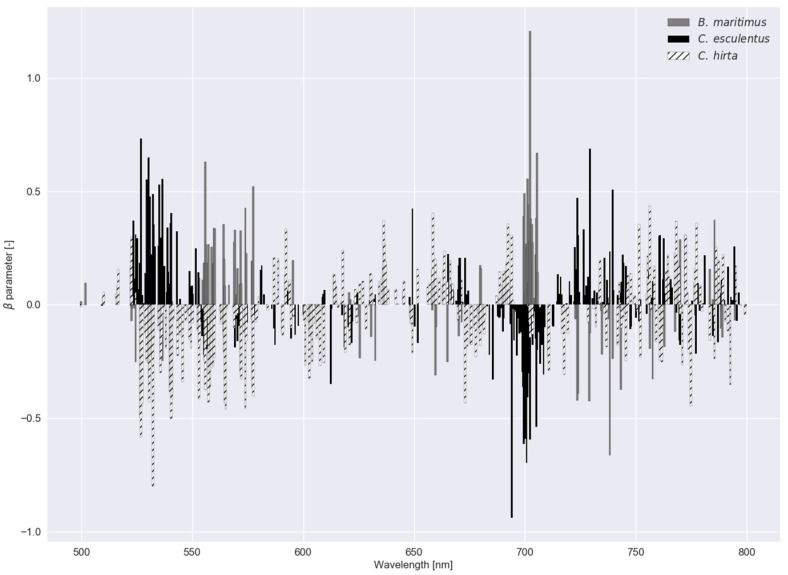
β parameter values for the regularized logistic regression with a dataset comprising reflectance data of *Cyperus esculentus*, *Bolboschoenus maritimus* and *Carex hirta* (Experiment I).

**Figure 7:**
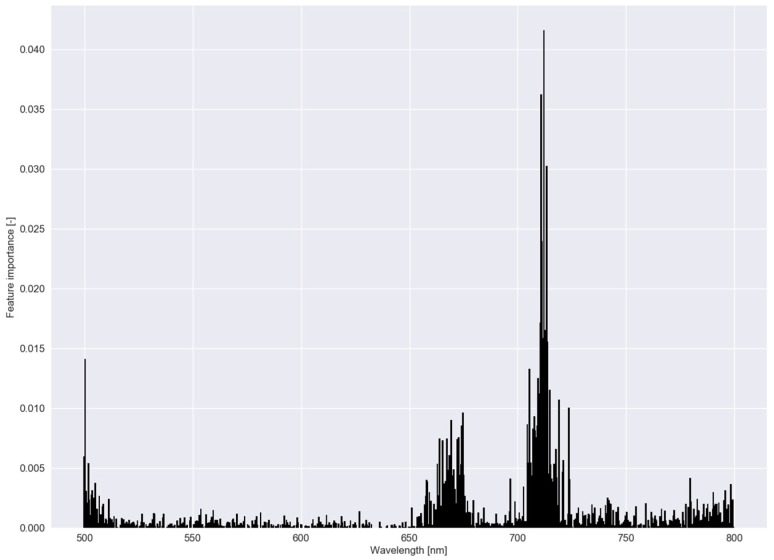
Feature importance of the random forest classifier using a dataset comprising reflectance data of *Cyperus esculentus, Bolboschoenus maritimus* and *Carex hirta* (Experiment I).

In Figure 6, β parameter values—a measure of feature importance—are depicted for each submodel in RLR of experiment I with the associated $C^{-1}$ values in Table 2. In experiment I, *B. maritimus* had the smallest $C^{-1}$ (or largest C), and hence, the most β parameter values that were reduced to zero, followed by *C. esculentus*. To distinguish *C. esculentus* from the other two species, approximately three band regions (around 540, 700 and 740 nm) were used in the model. In the red region, fewer β parameters were observed for *C. esculentus*. For the practical application it is important to distinguish *C. esculentus* from other weeds and not to distinguish between these morphologically similar weeds. For that reason, the β parameter values of *B. maritimus* and *C. hirta* are less important. In Figure 7, feature importance calculated using the RF model is shown, again, for experiment I. Here some bands in the red edge region were up to 40 times more important than bands in the green and NIR region. The red edge is defined as the sharp change in leaf reflectance between 680 and 750 nm [59]. RF also appoints feature around 500 nm and just before the red edge as important. RFs features are not able to reach zero as in RLR. Except for the importance of the red edge, no similarities between the feature importance strategies of the two models were observed. 

**Figure 8:**
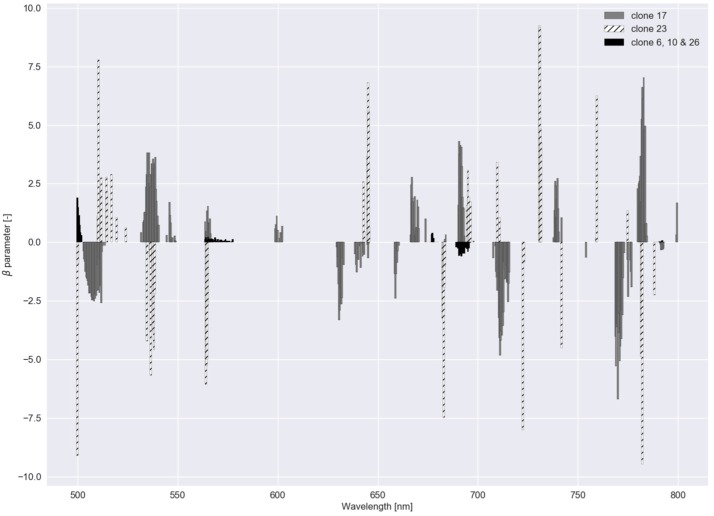
β parameters for the regularized logistic regression with a dataset comprising reflectance data of five clonal *C. esculentus* populations (Experiment II).

**Figure 9:**
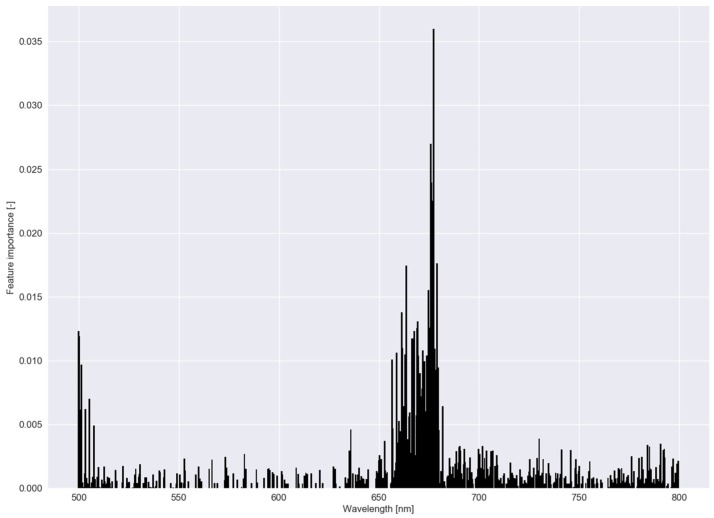
Feature importance of the random forest classifier using a dataset comprising reflectance data of five clonal *C. esculentus* populations (Experiment II).

These two graphs were also created for experiment II (Figure 8 and Figure 9). For the group of clone 6, 10 and 26, regularization strength was highest (Table 2), meaning a lower number of bands was used to distinguish between this group and the other clones. In Figure 9, feature importance, calculated with the RF model for Experiment II, shows an equal trend as in Experiment I. 

### 3.2. Robustness of the Models (Experiment III)

**Figure 10:**
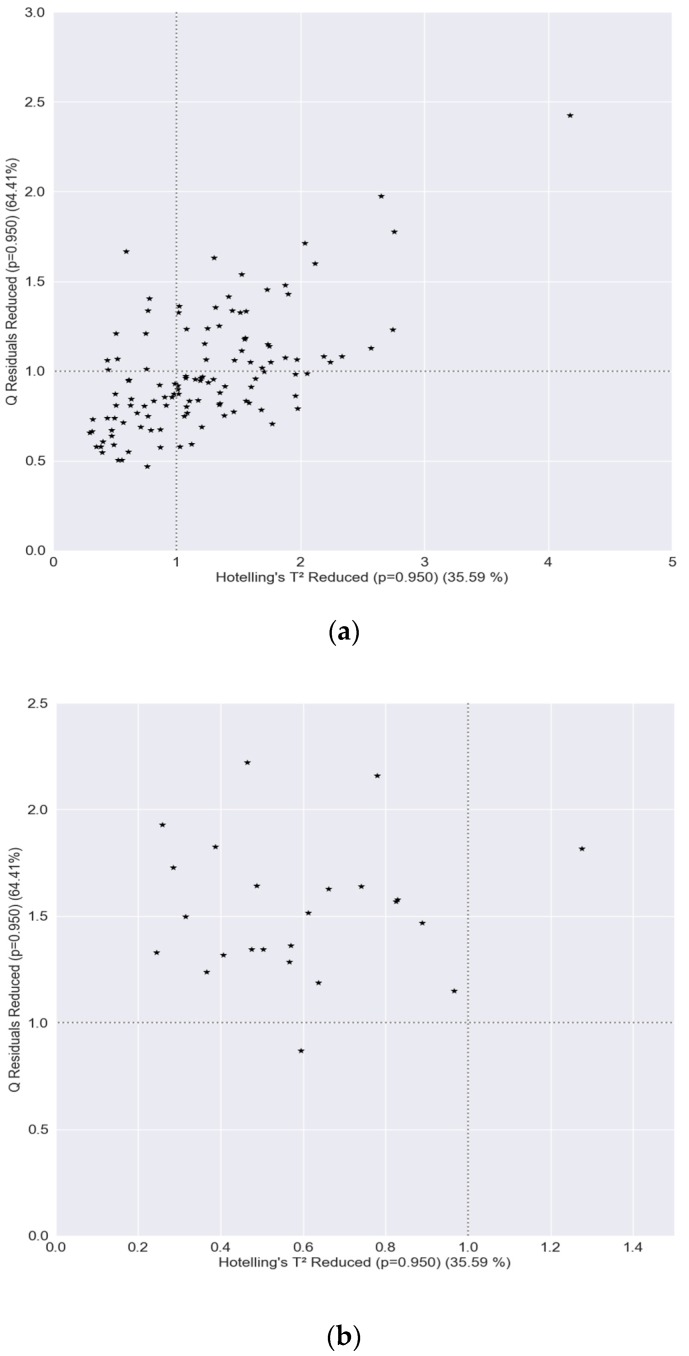
Reduced Q residuals plotted against Hotelling’s T²—reduced for (**a**) measurements from experiment II and for (**b**) samples collected at a maize field infested with *C. esculentus,* generated using the model from experiment I. Dotted grey lines indicate the 95% confidence limits.

Due to the availability of C. esculentus samples from different clonal populations and from an infested maize field in Lede, the robustness of the models built for experiment I could be tested (III). Putting those data in the three models produced varying results. On the one hand, RLR and PLS–DA were able to correctly classify all 120 *C. esculentus* samples from the dataset of experiment II and all 24 *C. esculentus* samples from the field in Lede. RF, on the other hand, classified only 16.67% and 4.17% correctly, respectively; 12 out of the 120 samples from experiment II were classified as *B. maritimus* and 88 as *C. hirta*; 8 out of 24 samples from the field in Lede were classified as *B. maritimus* and 15 as *C. hirta.* The RF model performed worst, as was the case in experiment I (Figure 4), The PLS-toolbox is able to automatically produce a graph of reduced Q residuals plotted against Hotelling’s T² Reduced. Therefore, this plot was made for PLS–DA of experiment I with the test data of experiment II and with the data of the field in Lede (Figure 10). Although all samples were correctly classified by the model, a great deal of the samples in (a) were labeled as outliers. This proves that these samples deviated from those obtained in experiment I but that the PLS–DA and RLR models were robust enough. To check the robustness of the model more extensively, intraspecific variability of *B. maritimus* and *C. hirta* should also be tested.

### 3.3. Low-Cost Tool (Experiment IV)

**Figure 11:**
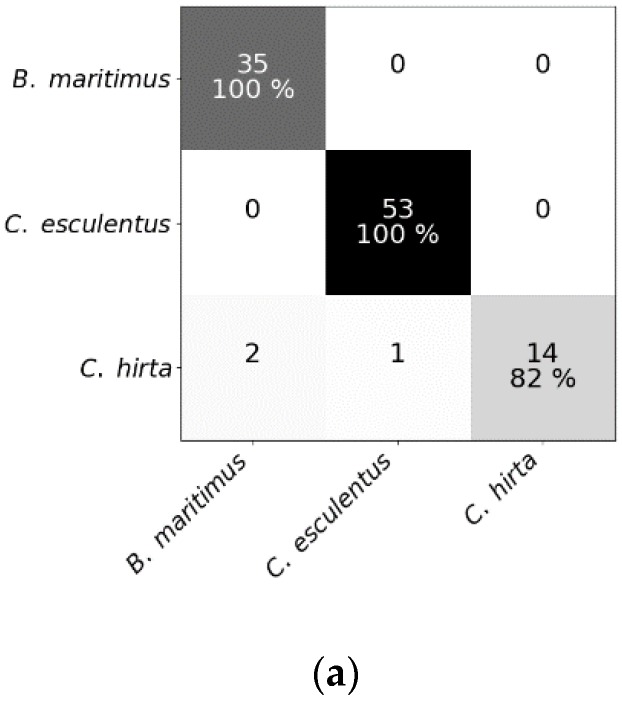

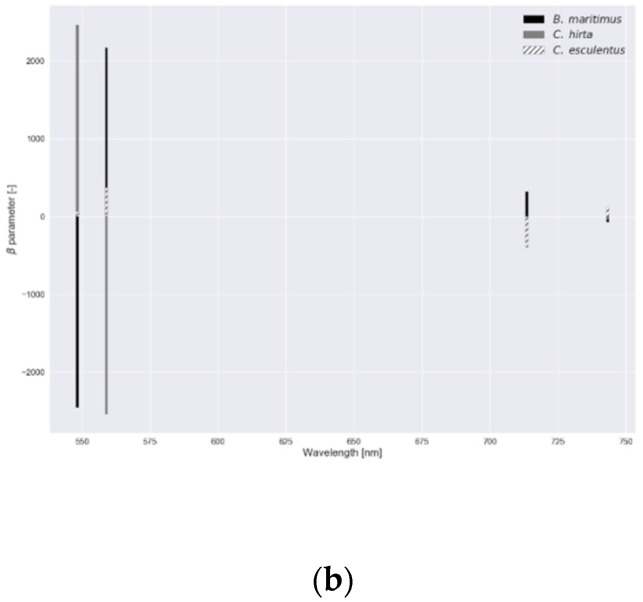
(**a**) confusion matrix and (**b**) β parameter values of a logistic regression with four spectral resampled bands of test data from experiment I.

**Figure 12:**
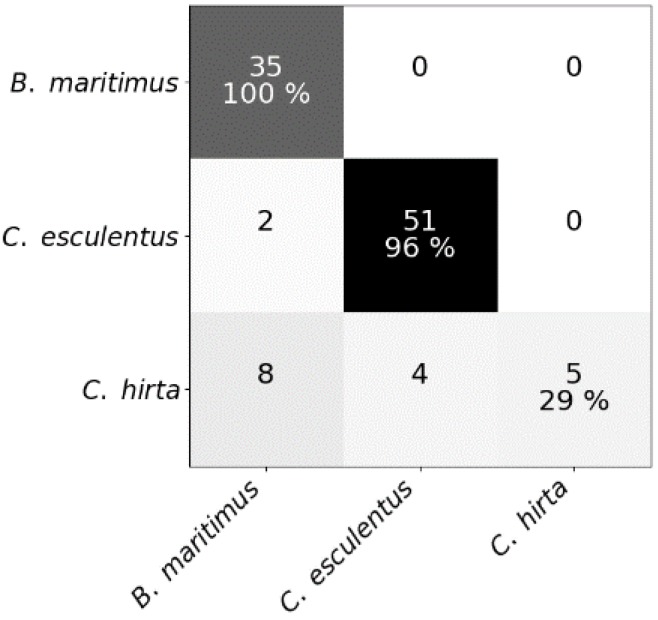
Confusion matrix of a logistic regression with four spectral bands, simulated from the Parrot Sequoia camera, created with the test dataset.

To test whether it would be possible to develop a small, portable device, sensitive in only some select regions of the electromagnetic spectrum, that could tell whether or not a farmer is dealing with *C. esculentus*, a new logistic regression was created. This logistic regression used four spectral bands that were selected based on a RLR with resampled data. Logistic regression was chosen as the model type because of the feature importance’s interpretability and because the regularized version outperformed RF in experiment I. Figure 11a shows the confusion matrix and Figure 11b the associated β parameter values. All samples of *C. esculentus* were correctly classified. One sample of *C. hirta* was classified as *C. esculentus* and two as *B. maritimus*. Only one extra sample was incorrectly classified (Figure 4). To increase classification certainty, farmers should therefore sample multiple leaves. Figure 12 shows the results of the logistic regression created with the simulated reflectance values of the Parrot Sequoia camera. Two samples of *C. esculentus* were misclassified as *B. maritimus* which could lead farmers to wrongly take no mitigation actions. Additionally, four samples of *C. hirta* were misclassified as *C. esculentus,* in comparison with one sample with the model with four customized bands. This comparison shows that there is a need for a specialized tool to discriminate between *C. esculentus* on the one hand and *C. hirta* and *B. maritimus* on the other. The authors propose a non-imaging tool with low spectral resolution and with a leaf clip. The objective is not to map all *C. esculentus* plants, but to check whether or not the weed is present. Because of *C. esculentus’* multiplying and spreading characteristics, it should suffice to measure a small number of weeds. Gutiérrez et al. [57] pointed out that building a low cost device based on a set of narrow-band wavelengths is not that straightforward and that the only way to obtain this subset is to measure the entire hyperspectral signature and discard the excess data. For their application, they managed to obtain only a slight reduction in performance when strongly reducing the numbers of bands by selecting the proper filters. For the detection of *C. esculentus* more research is needed, regarding filter creation and selection, in order to build a low-cost tool that is sensitive in a few discriminatory wavelengths.

## 4. Conclusions

The results showed that it is possible—using hyperspectral reflectance values—to discriminate between *C. esculentus,* and *C. hirta* and *B. maritimus*—two species often mistaken for *C. esculentus.* In this context, 103 out of 105 samples were correctly classified. Clones of *C. esculentus* that were genetically distinct, were also correctly classified using the same method. Here, 27 out of 30 samples were correct classified. Correct classification of clones may be relevant for *C. esculentus* control as efficacy of chemical control measures is clone-dependent. The robustness of the models that discriminated between *C. esculentus,* and *C. hirta* and *B. maritimus*, was checked by using the samples of different clonal populations of *C. esculentus* and *C. esculentus* plants collected in a maize field. Although the partial least squares–discriminant analysis model labeled some of the measurements as outliers, the model still classified them as *C. esculentus,* as did the regularized logistic regression model. The regularized logistic regression classifier appeared to be an appropriate model with which to classify *C. esculentus, C. hirta* and *B. maritimus,* and *C. esculentus* clones, and with which to appoint discriminatory wavelengths for the development of a specialized tool for in-field recognition of *C. esculentus*. More research is needed for the development of this tool. As weed identification skills vary among farmers and weeds grow in a wide range of outdoor conditions, such a tool should allow for the correct discrimination of *C. esculentus* plants from a broad range of co-occurring, visually-similar grass-like weeds, irrespective of environmental conditions. 

## Figures and Tables

**Table 1:** Explanation and values of the hyperparameters of the random forest classifiers.

Hyperparameter	Explanation	2018 (Exp. I)	2019 (Exp. II)
min_samples_split	Minimum numbers of samples required to split an internal node	3	5
min_samples_leaf	Minimum of samples required to be at a leaf node	1	1
max_features	Number of features to consider when looking for the best split (=l)	77	74

**Table 2:** Values for $C^{-1}$ for each ‘one versus rest’ submodel in RLR.

Submodel	2018 (Exp. I)	Submodel	2019 (Exp. II)
*B. maritimus*	8.50	Clone 17	291.51
*C. esculentus*	509.41	Clone 23	14.85
*C. hirta*	2718.59	Clone 6, 10 and 26	4.86
